# Supplementary material for: Preclinical Studies in Small Animals for Advanced Drug Delivery Using Hyperthermia and Intravital Microscopy
Source: Cancers (Basel). 2021 Oct 14;13(20):5146. doi: 10.3390/cancers13205146 (PMC8534089; doi:10.3390/cancers13205146)
Supplement: Supplementary file 1 [file cancers-13-05146-s001.zip › cancers-1376349-supplementary.pdf]

Supplementary Materials

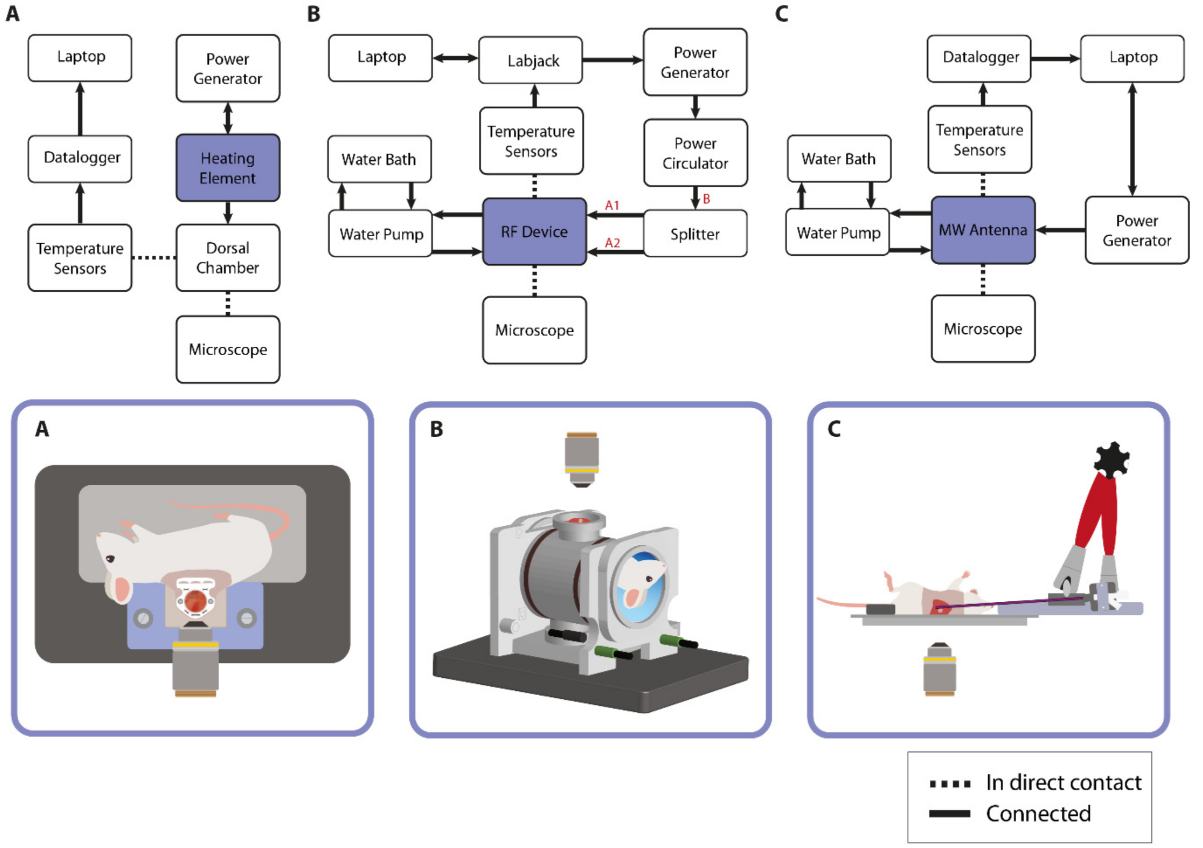

Figure S1. Schematic diagram of the advanced hyperthermia set-ups. A) The dorsal skinfold chamber; B) The cylindrical RF device; C) The directional microwave hyperthermia system.

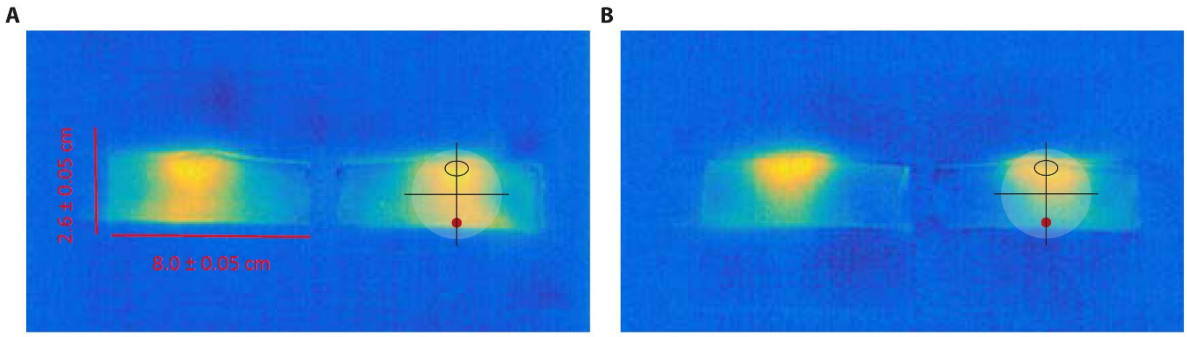

Figure S2. Thermographs of the heat distribution in a mouse shaped phantom. The x,z axis is drawn including the estimated position of a tumor (⬭) and the position of the central nervous system (●) in the model; A) Heating measurements performed without the addition of a salt water bolus (2.5 g/L) in the microscope viewing port indicate a large thermal zone throughout the entire phantom; B) Addition of the salted water bolus led to a more defined thermal zone, which was lifted upwards in comparison to panel A.

cancers

MDPI

Table S1. Overview of the hyperthermia devices for preclinical small animal studies in combination with intravital imaging

	**Dorsal Skinfold Chamber**		**Cylindrical RF Device**		**Directional Microwave Antenna**	
**Method**	Conduction		Radiofrequency (RF)		Microwave (MW)	
**Tumor Position**	Superficial<br>(Dorsal Skin)		Deep Seated<br>(Liver)		Superficial or Deep Seated<br>(Skin, Liver, Pancreas, Colon)	
**Setting**	External heating element temperature		433.92 MHz		2450 MHz	
**Device**	● Heating Element Temperature (°C)<br>● Treatment Duration (s)		● Power (W)<br>● Treatment Duration (s)		● Power (W)<br>● Treatment Duration (s)	
**Positioning**	● Chamber-to-stage holder		● Bore design		● TESA tape	
**Protection**	**Skin**	**Stage**	**Skin**	**Stage**	**Skin**	**Stage**
	-	-	● Water Bolus	-	● Ultrasound gel<br>● Active cooling applicator	● N/A
**Side-Effects**	● Hemorrhage		● Hot Spot Formation		● Hemorrhage	
**Advantages**	● Tumor Development via Optical Visualization<br>● Intravital Visualization<br>● Longitudinal Experiments		● Intravital Visualization<br>● Temperature Feedback Control		● Intravital Visualization<br>● Temperature Feedback Control	
**Disadvantages**	● High Technical Complexity<br>● Temperature Difference Tumor		● Vulnerable Device (*Water Bolus*)<br>● Prone to Development of Multiple SAR Foci		● High Technical Complexity<br>● Requires Invasive Surgery and Organ Manipulation<br>● Tuning of size SAR focus not yet included	
